# Effects of a solid lipid curcumin particle formulation on chronic activation of microglia and astroglia in the GFAP-IL6 mouse model

**DOI:** 10.1038/s41598-020-58838-2

**Published:** 2020-02-11

**Authors:** Faheem Ullah, Rustam Asgarov, Madhuri Venigalla, Huazheng Liang, Garry Niedermayer, Gerald Münch, Erika Gyengesi

**Affiliations:** 1 Department of Pharmacology, School of Medicine, Western Sydney University, Locked Bag 1797, Penrith, New South Wales 2751 Australia; 2 Department of Neurology, Shanghai Fourth People’s Hospital, Tongji University, Shanghai, China; 3 School of Science and Health, Western Sydney University, Locked Bag 1797, Penrith, New South Wales 2751 Australia; 4 NICM Health Research Institute, Western Sydney University, Locked Bag 1797, Penrith, New South Wales 2751 Australia

**Keywords:** Neurodegenerative diseases, Central nervous system infections

## Abstract

Chronic glial activation is characterized by increased numbers of activated glial cells, secreting free radicals and cytotoxic cytokines, subsequently causing neuronal damage. In order to investigate the anti-inflammatory activity of Longvida^®^ Optimised Curcumin (LC), we fed 500 ppm of LC to 2-month-old wild type and GFAP-IL6 mice for 6 months. LC feeding led to a significant reduction in the number of Iba-1^+^ microglia by 26% in the hippocampus and by 48% in the cerebellum, GFAP^+^ astrocytes by 30%, and TSPO^+^ cells by 24% in the hippocampus and by 31% in the cerebellum of the GFAP-IL6 mice. The morphology of the cells was assessed and LC significantly decreased the dendritic length of microglia and the convex area, convex perimeter, dendritic length, nodes and number of processes of astrocytes in the hippocampus while decreasing the soma area and perimeter in the cerebellum, in LC-fed GFAP-IL6 mice. In addition, LC feeding increased pre- and postsynaptic protein levels and improved balance measured by Rotarod. Together, these data suggest that LC is able to attenuate the inflammatory pathology and ameliorate neurodegeneration and motor deficits in GFAP-IL6 mice. For patients with neuro-inflammatory disorders, LC might potentially reverse the detrimental effects of chronic glial activation.

## Introduction

In a healthy brain, microglia and astrocytes play a key role in the normal function of the central nervous system (CNS). The inflammatory response is mediated by the activated microglia, which is considered as the hallmark of neuroinflammation. The chronic activation of microglia leads to neuronal damage through the release of various cytotoxic molecules such as cytokines, reactive oxygen intermediates, and proteinases^1^. Microglia are involved in neural development including through phagocytosis of apoptotic cells. They also influence the function of neurons and their progenitor cells^2^. Astrocytes regulate synaptic transmission, for example through re-uptake of neurontransmitters such as glutamate, and support neurons with metabolic substrates such as gluthathione and lactate^3^.

Chronic microglial activation, or neuroinflammation, has been described in many neurodegenerative diseases including chronic traumatic encephalopathy, amyotrophic lateral sclerosis, Parkinson’s (PD) and Alzheimer’s disease (AD)^4^. For sporadic AD, surmounting histological evidence points to chronic microglial activation as a part of the disease process^1,5^. Chronic microglial activation describes long lasting, CNS-specific, aberrant glial responses that do not reproduce the classic characteristics of inflammation although they do contribute to neurodegeneration^6^. To further support the role of neuroinflammation in AD, genome-wide association studies have identified a variety of inflammation-relevant genes that are associated with AD including clusterin (CLU), complement receptor 1 (CR1) and triggering receptor expressed on myeloid cells 2 (TREM2)^7^. The role of the microglial receptor TREM2 is to activate phagocytosis or microglial survival, and its link to AD is considered one of the strongest supporting arguments for the role of neuroinflammation in AD.

The progress of neuroinflammation is characterized by both increased microglia numbers and altered morphology. Under normal physiological conditions, microglia are designated as “resting” while the reactive morphology is termed “activated.” Under physiological conditions, microglial shows long, delicate branched processes oriented radially to a small elliptical soma. However, they are never in a real resting state and play a critical constitutive role in scanning the environment^8^. In diseased tissue, due to injury or neuroinflammatory stimuli, microglia appear quite different, and go into an “activated state” with enlarged cell bodies, shortened and fewer processes and, a phagocytic appearance. Apoptotic cells, lipopolysaccharide, inflammatory cytokines or aggregated proteins could trigger microglial cells *in vitro* to retract their processes and adopt amoeboid-like morphologies sometimes termed as “de-ramified microglia”^9–11^. While an acute microglia response can often resolve, under diseased conditions, microglia can remain in permanent chronic activation.

To histologically identify microglia, some of the most commonly used microglia markers are Iba-1 (ionized calcium binding adaptor molecule 1) and TSPO (translocator protein 18 kDa). Iba-1 is a microglia/macrophage specific calcium binding protein, while TSPO is a mitochondrial translocator protein predominantly expressed in the microglia, astrocytes, and macrophages in the blood vessels in the nervous system, specifically in the outer mitochondrial membrane of injured or activated cells^12–14^.

Astrocytes are the most abundant cell type of the CNS, playing a major role in brain homeostasis, providing metabolites and growth support to neuron synapse formation and plasticity and regulating the extracellular balance of ions and fluids^15^. Astrocytes are activated in response to neural insults, such as neuroinflammation and release cytokines, ILs, NO, and other potentially cytotoxic molecules^16,17^. Astrocytes respond to CNS challenges and the activated astrocytes are a hallmark of neuroinflammation which is characterized by high-level expression of glial fibrillary acidic protein (GFAP)^18^. Astrocytes display a bushy or spongiform morphology with very fine processes^19^, and when activated, they exhibit striking increases in GFAP immunoreactivity and in the number and length of GFAP-positive processes^20^.

In this study, we have used the GFAP-IL6 mouse which has been shown to be an established model of chronic glial activation and subsequent neurodegeneration. CNS-targeted production of pro-inflammatory cytokines including IL-1^21^, IL-6^22^ or TNF-α^23^ is a controlled way to initiate chronic glial activation. In the GFAP-IL6 mouse model, the trigger is the brain specific production of the cytokine IL6 by astrocytes (GFAP-IL6), resulting in both microglia and astroglia activation. Campbell *et al*., observed impairment of motor function and cognitive skills in hemizygous GFAP-IL6 mice at 12 months (homozygous GFAP-IL6 mice have a life span of <12 months), with cognitive decline, that were tested in a “conditioned avoidance response in a discriminated Y-maze” task^24^.

Our earlier investigation has established that in the GFAP-IL6 mice, fine motor skills already deteriorate from 6 months of age on, accompanied by significantly increased micro- and astroglia numbers in both the cerebellum and the hippocampus^25^. In addition, synaptic deficits (synaptophysin, PSD95) were also observed in GFAP-IL6 mice compared to the WT mice from 14 months onwards, indicating impaired synaptic transmission^25^. The aim of this study was to investigate the effects of LC on microglia and astroglia numbers as well as the glial morphology in the GFAP-IL6 mouse, in parallel with motor function.

To test if chronic neuroinflammation could be reduced by pharmacotherapy, in this study, we used the main ingredient of the spice turmeric (*Curcuma longa*), curcumin, which is a cytokine suppressive anti-inflammatory drug (CSAID). CSAIDs have a broad range of anti-inflammatory effects and target the pro-inflammatory AP1 and NF-κB signaling pathways and inhibit the expression of many pro-inflammatory cytokines in the low µM range^26^. Curcumin was shown to down-regulate the expression of cyclooxygenase-2 (COX-2), inducible nitric oxide synthase (iNOS), TNF-α, IL-1, -2, -6, -8, and -12. It inhibits IL-6 mediated signalling via inhibition of IL-6 induced STAT3 phosphorylation and consequent STAT3 nuclear translocation^27^, and interferes with the first signalling steps downstream of the IL-6 receptor in microglial activation^4^. To improve the bioavailability of curcumin, highly bioavailable curcumin formulations (encapsulated in lipid-based matrices such as liposomes and micelles) have been developed. One of these formulations is Longvida^®^ Optimised Curcumin (LC) (Verdure Sciences Inc.) which can achieve µM concentrations in the rodent brain^28,29^. Human clinical trial data indicate that curcumin might exert effects in the CNS, but evidence for its effects on chronic microglial and astroglia activation in the CNS are still limited.

This study set out to evaluate the effects of LC on the numbers and morphology of both astrocytes and microglia as well as markers of synaptic connectivity and motor function in the GFAP-Il6 mice.

## Results

### LC did not significantly affected the rate of body weight gain of the mice

**Figure 1:**
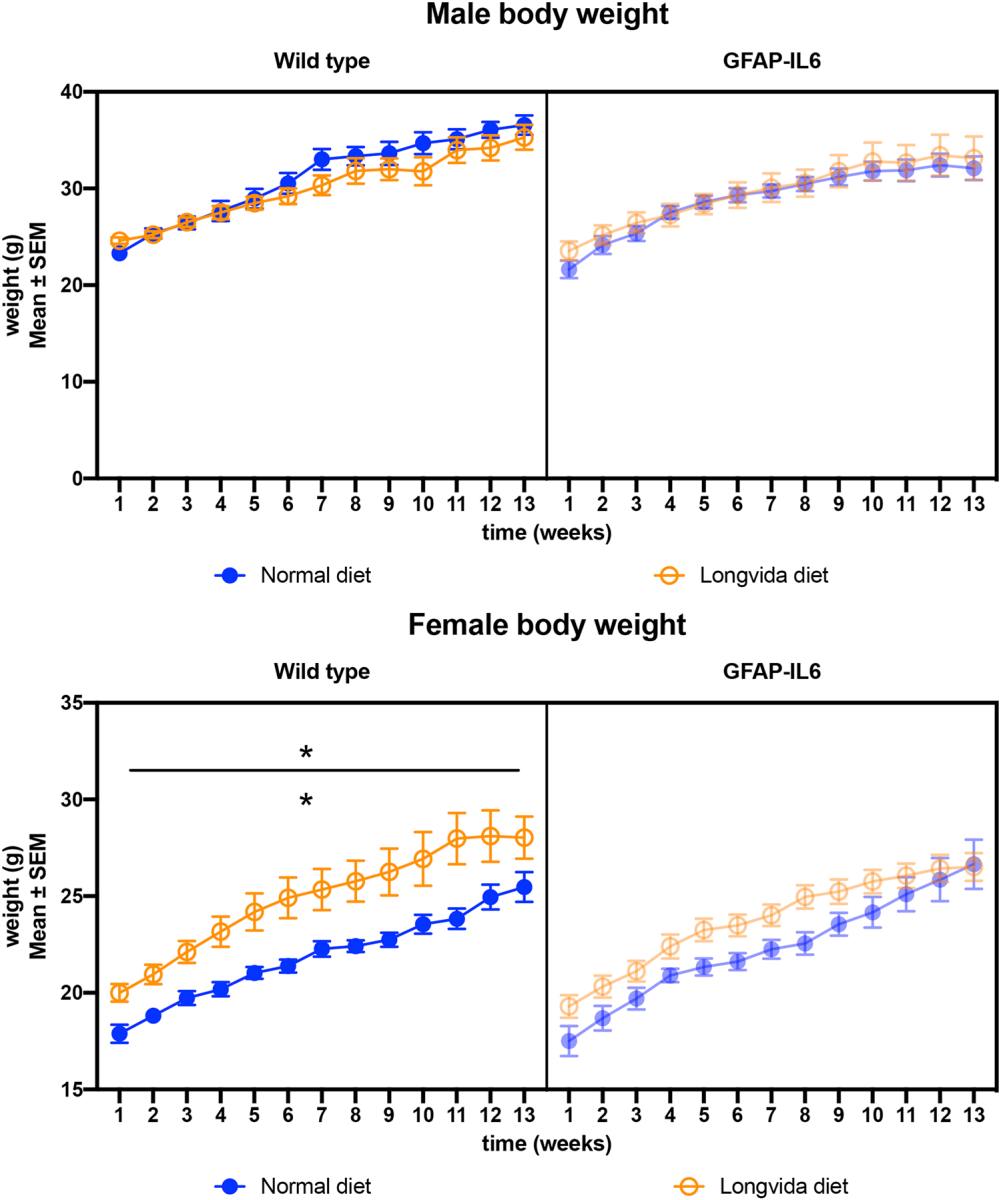
Weight change of animals during the 26 weeks of feeding. (**A**) Weight change of male mice showed no significant differences between groups. (**B**) We found significant (*p* = 0.006) differences between the wild type normal and LC diet female groups, with the LC fed animals being heavier. The weight of the GFAP-IL6 animals was not significantly affected by diet.

To make sure that the animals were consuming enough LC containing pellets and gaining weight throughout the study, we followed their body weight weekly for 13 weeks. We found no significant changes in the body weight of the male mice between genotypes and diets, using a three-way ANOVA analysis. The animals were definitely gaining weight, supported by the significant time effect [F(1.712, 47.94) = 147.0, *p* < 0.0001], while diet [F(1, 28) = 1.86, *p* = 0.182] or genotype [F(1, 28) = 0.042, *p* = 0.83] alone did not have a significant effect on the body weight of the animals (Fig. 1A). We also found a significant interaction between time and diet [F(12, 336) = 2.53, *p* = 0.003] for the male animals.

The body weight of females was analyzed separately by a three-way ANOVA as well. We found also a strong effect of time [F(2.208, 79.48) = 169.1, *p* < 0.0001] on the weight gain of the animals, however, we also noticed a significant effect of genotype [F(1, 36) = 12.46, *p* = 0.001], with no interaction between these variables. Further, we investigated the different genotype groups, using two-way ANOVA, and found in the case of the wild type females, the diet had a significant effect [F(1, 18) = 9.57, *p* = 0.006], resulting in slightly higher body weight throughout the study (Fig. 1B).

### LC significantly decreased the Iba-1^+^ microglia numbers in the hippocampus and cerebellum

In order to test the genotype differences between wild type (WT) and GFAP-IL6 normal food-fed mice, and the effect of LC on the microglial number on both wild type and GFAP-IL6 mice, immunohistochemistry and stereological counting of Iba-1^+^ microglia were performed in the hippocampus and cerebellum in control and LC-fed cohorts.

**Figure 2:**
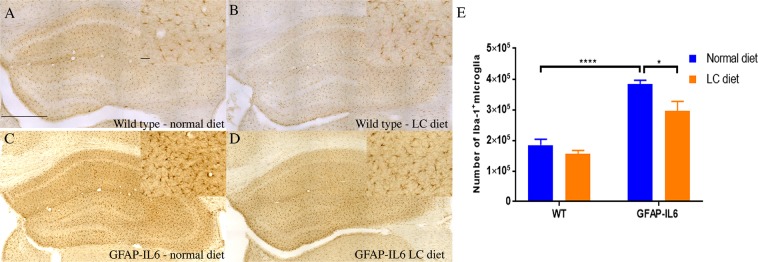
Representative photomicrographs of immunohistochemical staining of the hippocampus showing decreased numbers of microglia (Iba-1^+^ cells) in the GFAP-IL6 LC-fed mice compared to their regular diet-fed counterparts. (**A**–**D**) Images are representative of at least six animals per group (n = 6). Magnification 10× objective field, scale bar = 500 µm and 100 µm in inserts. (**E**) Graphical representation of total Iba-1^+^ count in the hippocampus. The graph represents the mean ± SEM and significant differences were determined using a two-way ANOVA (**p < 0.0015).

Using two-way analysis of variance (ANOVA) on our cohorts, a significant difference was found between the groups [F(3,16) = 35.10, *p* < 0.0001] in the hippocampus, where the wild type mice had 184,706 ± 19,037 Iba-1^+^ microglia, and wild type LC-fed mice had slightly (16.53%) less (156,498 ± 10,762) **(**Fig. 2A,B,E). In contrast, the GFAP-IL6 mice had 383,588 ± 12,253 Iba-1^+^ microglia, which was significantly reduced to 295,666 ± 31,017 (25.88%) in LC-fed mice (*p* < 0.02) **(**Fig. 2C–E).

**Figure 3:**
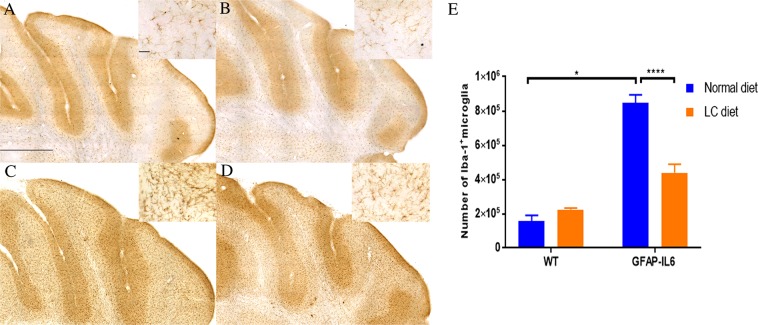
Representative photomicrographs of immunohistochemical staining for the microglia (Iba-1^+^ cells) of the cerebellum showing the LC feeding has significantly reduced the number of microglia in the GFAP-IL6 LC-fed animals. (**A**–**D**) (Magnification 10× objective field, scale bar = 500 µm and 100 µm in inserts). Images are representative of at least six animals per group (n = 6). (**E**) Graphical representation of total Iba-1^+^ count in the cerebellum. The graph represents the mean ± SEM and statistical analyses were applied using a two-way ANOVA (****p < 0.0001, **p < 0.05).

In the cerebellum, a significant difference was found between the groups [F(3,23) = 16.72, *p* < 0.0001]. The wild type mice had 156,454 ± 33,721 Iba-1^+^ microglia which was slightly, but not significantly increased in wild type LC-fed mice (222,026 ± 7,555) **(**Fig. 3A,B,E). The GFAP-IL6 mice had 847,456 ± 46,120 Iba-1^+^ microglia, which was significantly downregulated (by 48%) in LC-fed GFAP-IL6 mice (439,038 ± 49,423) (*p* < 0.0001) **(**Fig. 3C–E).

### LC downregulated the number of TSPO^+^ cells in GFAP-IL6 mice in the cerebellum but not in the hippocampus

TSPO is a translocator protein expressed on the outer mitochondrial membrane. It is increased in response to injury and inflammation^30^. In order to investigate the genotype difference between the wild type and GFAP-IL6 normal food-fed mice, and to test the effect of LC on TSPO^+^ microglia in both wild type and GFAP-IL6 mice, immunohistochemistry and stereological counting of the TSPO positive microglia/macrophages was performed.

**Figure 4:**
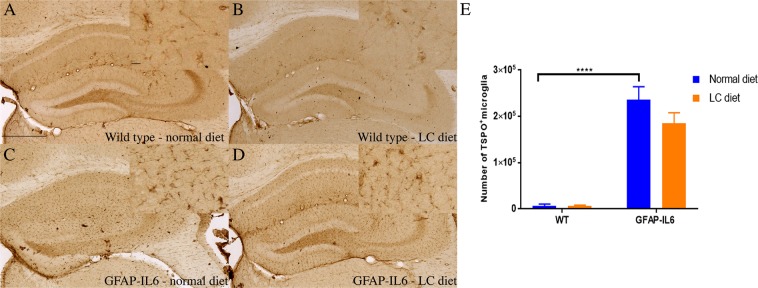
Representative photomicrographs of immunohistochemical staining for the microglia (TSPO^+^ cells) of the hippocampus showing that LC feeding has significantly increased the number of TSPO^+^ microglia in the GFAP-IL6 LC normal-fed animals. (**A**–**D**) (Magnification 10× objective field, scale bar = 500 µm and 100 µm in inserts). Images are representative of at least six animals per group (n = 6). (**E**) Graphical representation of total TSPO^+^ count in the hippocampus. The graph represents the mean ± SEM and the percentage differences were determined using a two-way ANOVA (*p* < 0.19).

In the hippocampus, a significant difference was observed between the groups [F(3,10) = 30.80, *p* < 0.0001]. The wild type mice had 6,626 ± 3,952 TSPO^+^ microglia, which was comparable to the numers of the LC-fed wild type mice (6,755 ± 1,730) **(**Fig. 4A,B,E). In contrast, GFAP-IL6 normal fed mice had 236,098 ± 28,005 TSPO^+^ microglia, which were reduced by 24.46% in LC-fed GFAP-IL6 mice (184,624 ± 23,174) (*p* > 0.1) **(**Fig. 4C–E).

**Figure 5:**
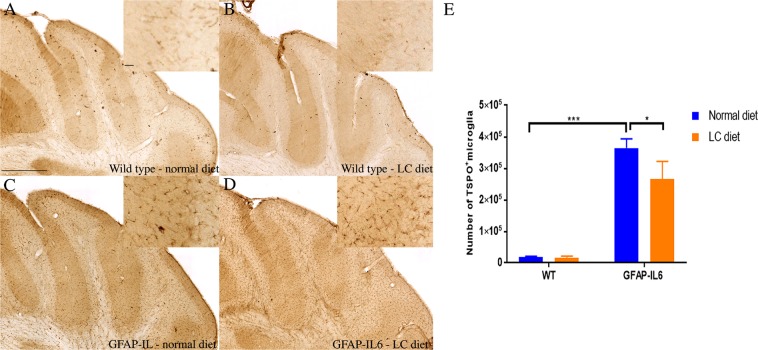
Representative photomicrographs of immunohistochemical staining for the microglia (TSPO^+^ cells) of the cerebellum showing that LC feeding has significantly reduced the number of TSPO^+^ microglia in the GFAP-IL6 LC-fed animals. (**A**–**D**) (Magnification 10× objective field, scale bar = 500 µm and 100 µm in inserts). Images are representative of at least six animals per group (n = 6). (**E**) Graphical representation of total TSPO^+^ count in the cerebellum. The graph represents the mean ± SEM and significant differences were determined using a two-way ANOVA (***p < 0.001, *p < 0.05).

In the cerebellum, a significant differenence was found between the groups [F(3,10) = 23.89, *p* < 0.0001]. Wild type mice had 18,108 ± 2,933 TSPO^+^ microglia, which were only slightly decreased by 5.73% in LC-fed wild type mice (17,098 ± 4,161) (*p* > 0.1) (Fig. 5A–B,E). GFAP-IL6 mice on a normal diet had 364,942 ± 28,577 TSPO^+^ cells, which was significantly downregulated by 31.37% in the LC-fed GFAP-IL6 group (265,979 ± 55,791) (*p* < 0.005) (Fig. 5C–E). The stereological data of both Iba-1 and TSPO in the hippocampus and the cerebellum is summarized in Table 1.

**Table 1:** Stereological counting summary of the Iba-1, TSPO positive microglia in the hippocampus and the cerebellum. All values are presented as mean ± SEM.

Genotype	Diet	Iba-1 hippocampus	Iba-1 cerebellum	TSPO hippocampus	TSPO cerebellum	GFAP hippocampus
Wild type	Normal food	184,706 ± 19,037	156,454 ± 33,721	6,626 ± 3,952	18,108 ± 2,933	211,968 ± 11,730
Wild type	LC 500 ppm	156,498 ± 10,762	222,026 ± 7,555	6,755 ± 1,730	17,098 ± 4,161	161,441 ± 19,648
GFAP-IL6	Normal food	383,588 ± 12,253	847,456 ± 46,120	236,098 ± 28,005	364,942 ± 28,577	372,850 ± 36,861
GFAP-IL6	LC 500 ppm	295,666 ± 31,017	439,038 ± 49,423	184,624 ± 23,174	265,979 ± 55,791	264,105 ± 24,115

### LC decreased the number of GFAP^+^ astrocytes in the hippocampus

In order to quantify the number of astrocytes, GFAP immunostaining and stereological quantification were performed to investigate the genotype difference between wild type and GFAP-IL6 normal food-fed mice. Furthermore, the effect of LC on the elevated number of GFAP^+^ astrocytes in the wild type GFAP-IL6 mice was investigated in the hippocampus and cerebellum.

**Figure 6:**
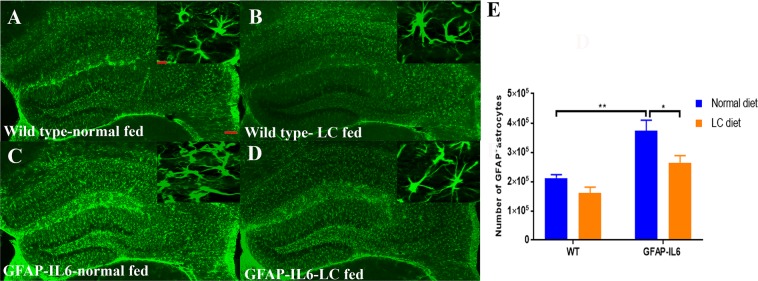
Representative photomicrographs of immunofluorescence staining for the astrocytes (GFAP^+^ cells) in the hippocampus showing the LC feeding has significantly reduced the number of GFAP^+^ astrocytes in the GFAP-IL6 LC-fed animals. (**A**–**E**) (Magnification 5× objective field, scale bar = 100 µm in 20× and 20 µm). Images are representative of at least six animals per group (n = 6). (**E**) Graphical representation of total GFAP count in the hippocampus. The graph represents the mean ± SEM and significant differences were determined using a two-way ANOVA (*p < 0.05, **p < 0.001).

In the hippocampus, GFAP-IL6 mice had a significantly larger number of GFAP^+^ astrocytes (372,850 ± 36,861) compared to wild type mice (211,968 ± 11,730) (*p* < 0.0016) **(**Fig. 6A,C). In LC-fed wild type mice, the number of astrocytes reduced by 23% (161,441 ± 19 648) compared to their normal diet fed counterparts. Whereas, in LC-fed GFAP-IL6 mice, a significant reduction (30%) in GFAP^+^ astrocytes was observed (264,105 ± 24,115) compared to the GFAP-IL6 normal food-fed mice **(**Fig. 6A–D,E).

**Figure 7:**
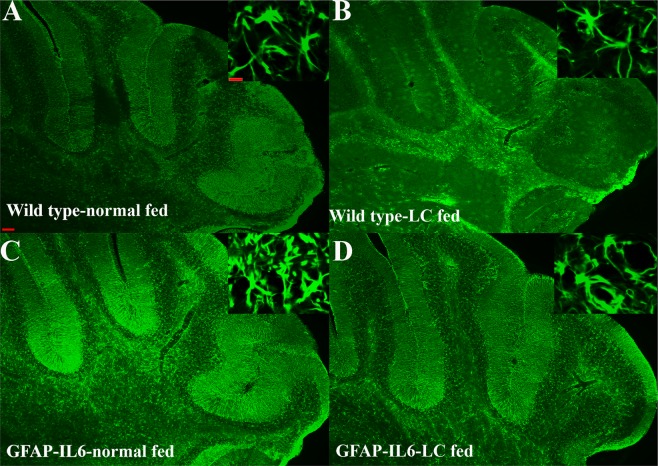
Representative photomicrographs of immunofluorescence staining for the astrocytes (GFAP^+^ cells) in the cerebellum showing that LC-fed groups appeared to have a somewhat lower expression of GFAP protein compared to GFAP-IL6 group. (Magnification 5× objective field, scale bar = 100 µm in 5× and 20 µm in inserts). (**A**–**D**) Images are representative of at least six animals per group (n = 6).

In the cerebellum, the astrocytes and their processes were very closely packed, making stereological counting difficult, hence the numbers of GFAP^+^ cells were not serologically analyzed. However, high-level expression of GFAP protein was observed in GFAP-IL6 mice compared to wild type mice, located mostly but not exclusively in the gray matter of the cerebellum, with a strong presence of elongated microglia in the white matter and molecular layer. The difference in the distribution of astrocytes between the wild type and GFAP-IL6 animals was substantial. Using qualitative observation, LC-fed groups appeared to have a comparatively low expression of GFAP protein compared to the GFAP-IL6 group (Fig. 7).

### Effect of LC on microglial morphology in the hippocampus and cerebellum

In neuroinflammation or after brain injury, a de-ramification of microglia has been observed, in which the microglia enter an “activated state” characterized by swollen ramified cells with some larger and some shorter dendrites^10^. In order to investigate the genotype difference and the effect of the LC diet on Iba-1^+^ microglial cells, we analyzed the morphological features of the Iba-1^+^ microglia in the hippocampus and the cerebellum of the wild type and GFAP-IL6 mice, for both the normal-fed and LC-fed animals.

**Figure 8:**
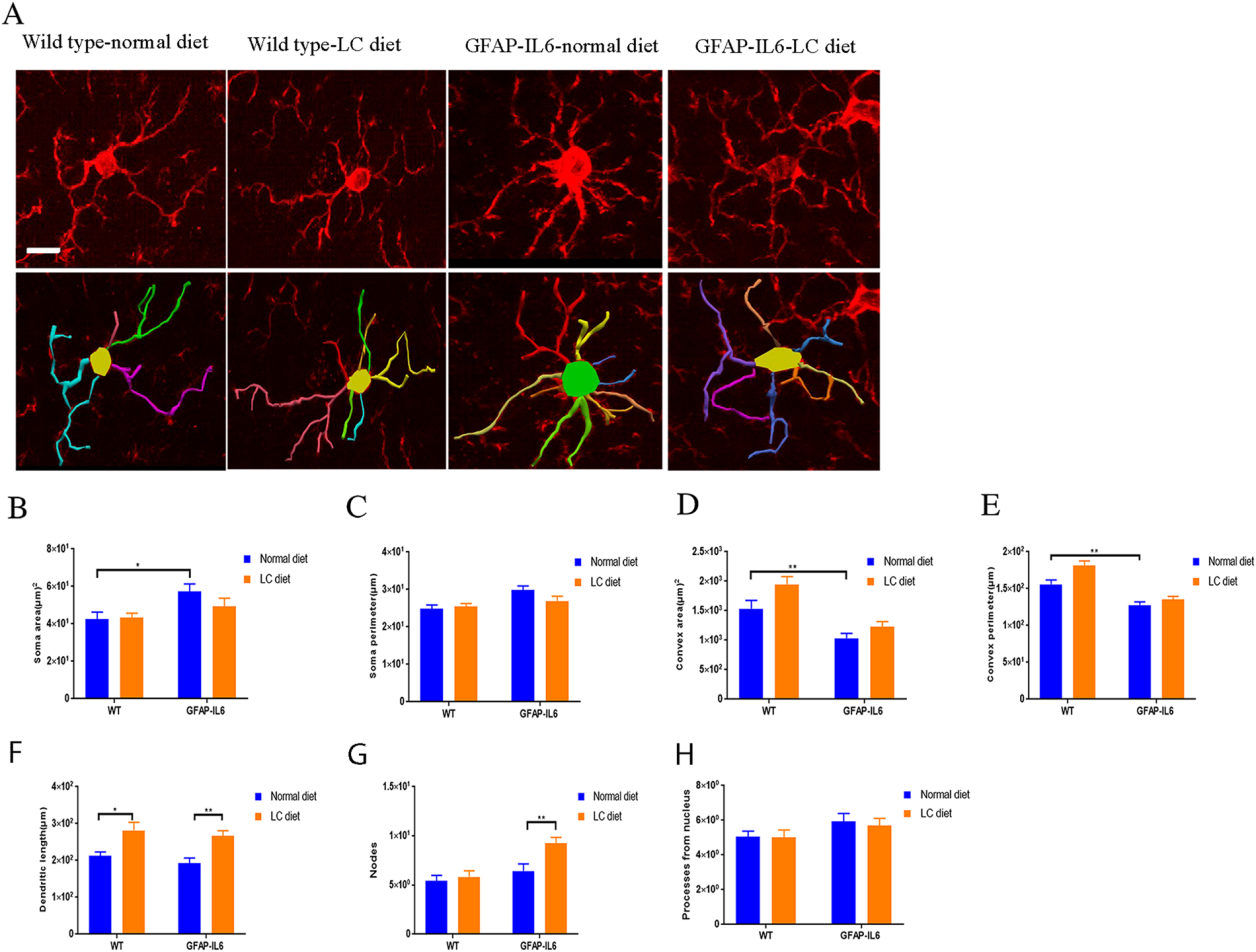
Effect of LC on microglial morphology in the hippocampus. (**A**) The representative images of microglia immunostained for Iba-1 in the hippocampus showing that the Iba-1^+^ microglial cells of GFAP-IL6 LC-fed mice had a significantly reduced soma area, soma perimeter and a higher number of nodes than that of GFAP-IL6 normal-fed mice. Scale bar 10 µm. (**B**–**H**) Graphs showing the morphological changes in Iba-1^+^ microglia. Two-way ANOVA, Tukey’s post-test, mean ± SEM (*p < 0.05, **p < 0.001).

In the hippocampus, a difference in a few parameters between the genotypes was observed, as the Iba-1^+^ microglial cells of GFAP-IL6 normal-fed mice had significantly larger soma areas (57.25 ± 4.03 µm^2^) (*p* < 0.04), with smaller convex areas (1035.60 ± 79.12 µm^2^) (*p* < 0.0008) and convex perimeters (127.68 ± 4.39 µm) (*p* < 0.001) compared to those of wild type [soma areas (42.57 ± 3.82 µm^2^), convex areas (1535.63 ± 139.68 µm^2^) and convex perimeter (155.83 ± 6.11 µm)]. In addition, Iba-1^+^ microglia in wild type LC-fed mice had significantly larger dendritic length (281.94 ± 21.97 µm) (*p* < 0.014) than those of the wild type normal-fed mice (212.90 ± 10.23 µm). However, the Iba-1^+^ microglial cells of GFAP-IL6 LC-fed mice had a significantly larger dendritic length (266.83 ± 14.10) (*p* < 0.003) and number of nodes (9.25 ± 0.59) (*p* < 0.003) than that of GFAP-IL6 normal-fed mice [Dendritic length (192.16 ± 14.58), number of nodes (6.43 ± 0.70)] (Fig. 8 A–H, Table 2).

**Table 2:** Morphological analysis of Iba-1^+^ microglia in the hippocampus (H) and the cerebellum (C).

Genotype	Diet	Soma area (µm)^2^		Soma perimeter (µm)		Convex 2D (area) (µm)^2^		Convex perimeter		Total length of dendrites		Number of Nodes		Number of Dendrites	
		H	C	H	C	H	C	H	C	H	C	H	C	H	C
Wild type	Normal food	42.5 ± 3.8	38.8 ± 2.8	24.8 ± 1.0	24.1 ± 0.8	1535.6 ± 139.6	1750.7 ± 148.9	155.8 ± 6.1	171.4 ± 7.9	212.9 ± 10.2	199.5 ± 14.7	5.4 ± 0.5	4.2 ± 0.5	5.0 ± 0.3	3.6 ± 0.3
Wild type	LC food	43.4 ± 2.3	40.2 ± 2.2	25.5 ± 0.7	24.1 ± 0.7	1946.1 ± 132.5	2038.3 ± 191.5	181.9 ± 5.6	180.2 ± 8.5	281.9 ± 21.9	265.3 ± 21.5	5.8 ± 0.6	4.7 ± 0.7	5.0 ± 0.4	3.7 ± 0.2
GFAP-IL6	Normal food	57.2 ± 4.0	77.6 ± 7.8	29.8 ± 1.1	37.2 ± 2.4	1035.6 ± 79.1	1087.9 ± 150.0	127.6 ± 4.3	137.5 ± 9.4	192.1 ± 14.5	169.1 ± 17.5	6.4 ± 0.7	4.9 ± 0.6	5.9 ± 0.4	4.3 ± 0.3
GFAP-IL6	LC food	49.2 ± 4.4	50.1 ± 4.7	26.9 ± 1.2	28.1 ± 1.6	1230.8 ± 86.0	1348.8 ± 127.2	135.3 ± 4.2	151.1 ± 7.5	266.8 ± 14.1	212.3 ± 14.8	9.2 ± 0.5	8.0 ± 1.0	5.6 ± 0.4	3.6 ± 0.3

**Figure 9:**
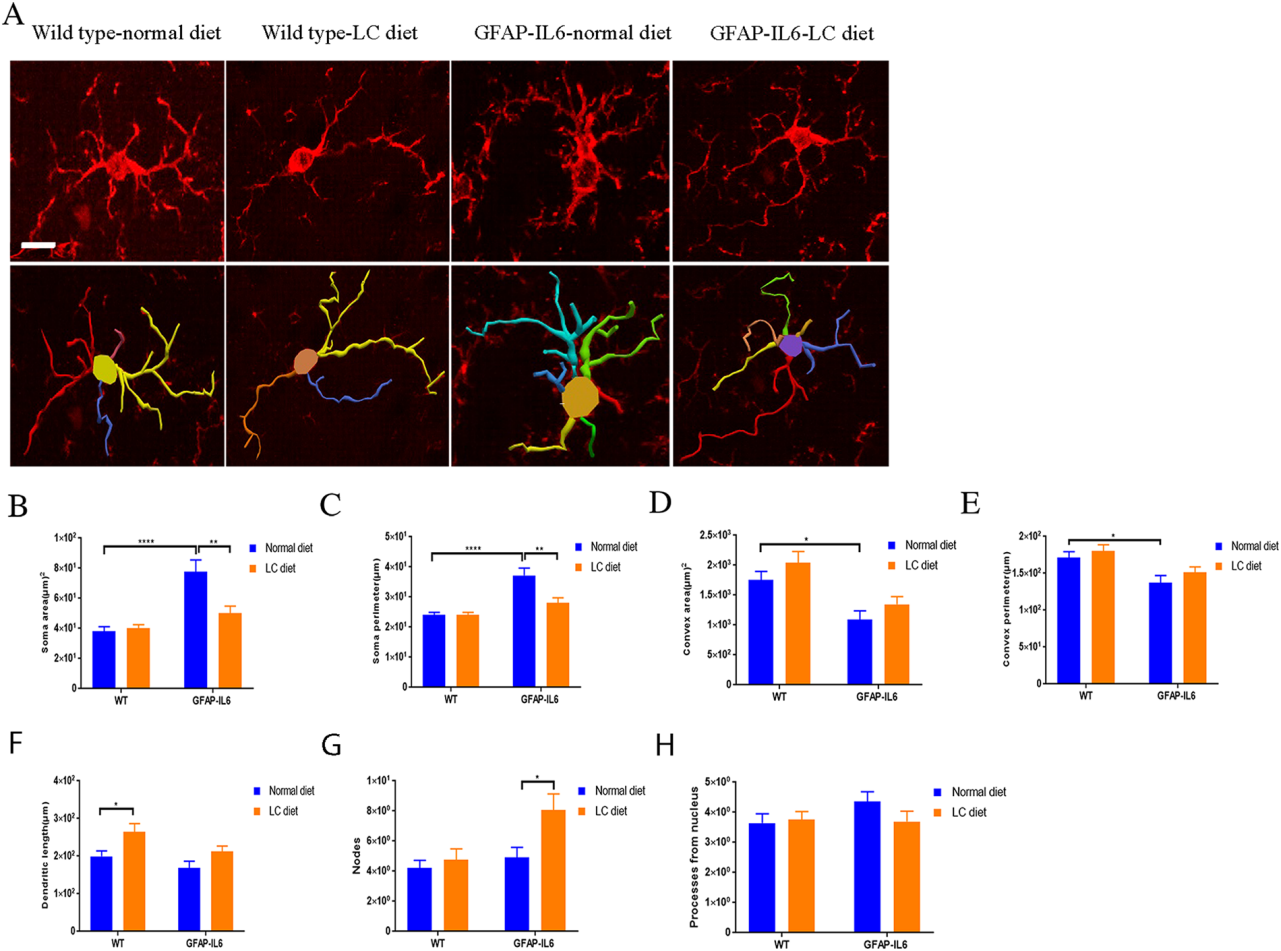
Effect of LC on microglial morphology in the cerebellum. (**A**) The representative images of microglia immunostained for Iba-1^+^ in the cerebellum showing that GFAP-IL6 LC-fed mice had a significantly reduced soma area, soma perimeter and a higher number of nodes than that of GFAP-IL6 normal-fed mice. (Scale bar 10 µm). (**B**–**H**) Graphs showing the morphological changes in Iba-1^+^ microglia. Two-way ANOVA, Tukey’s post-test, mean ± SEM (*p < 0.05, **p < 0.001, ****p < 0.0001).

In the cerebellum, a significant difference was observed in the genotype as the Iba-1^+^ microglial cells of GFAP-IL6 mice normal-fed had a significantly larger soma area (77.68 ± 7.89 µm^2^) (*p* < 0.0001), soma perimeter (37.21 ± 2.44 µm) (*p* < 0.0001) and a smaller convex area (1087.96 ± 15 µm^2^) (*p* < 0.015) and convex perimeter (137.57 ± 9.45 µm) (*p* < 0.026) compared to those of the wild type normal-fed mice [soma area (38.82 ± 2.80 µm^2^), soma perimeter (24.10 ± 0.82 µm) and small convex area (1750.71 ± 148.98 µm^2^), convex perimeter (171.42 ± 7.98 µm). Also, the Iba-1^+^ microglia in the wild type LC diet had a significantly larger dendritic length (265.30 ± 21.52 µm) (*p* < 0.048) than wild type normal-fed mice (199.55 ± 14.70). However, the Iba-1^+^ microglial cells of GFAP-IL6 LC-fed mice had a significantly reduced soma area (50.18 ± 4.77 µm^2^) (*p* < 0.002), soma perimeter (28.10 ± 1.68 µm) (*p* < 0.001) and a higher number of nodes (8.07 ± 1.06) (*p* < 0.024) compared to that of GFAP-IL6 normal-fed mice [soma area (77.68 ± 7.89 µm^2^), soma perimeter (37.21 ± 2.44 µm) number of nodes (4.92 ± 0.65)] (Fig. 9A–H, Table 2).

**Figure 10:**
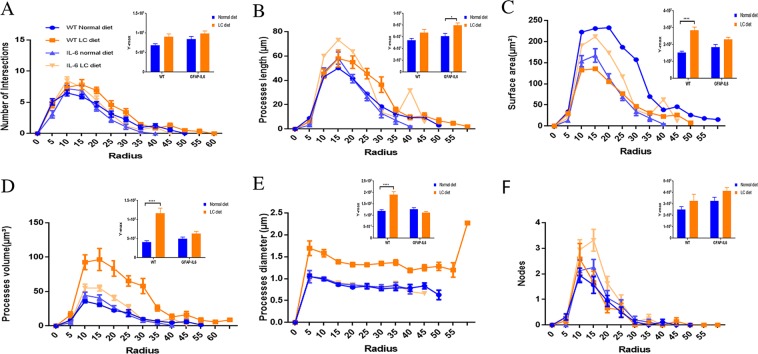
The effect of LC on microglial cells in Sholl analysis in the hippocampus region. (**A**–**F**) The representative graphs showing that LC-fed GFAP-IL6 mice have significantly larger processes in length compared to GFAP-IL6 normal-fed mice and no significant difference between wild type and GFAP-IL6 normal-fed mice in any parameter. wild type LC-fed mice have a significantly larger surface area, process volume, and process diameter than that of wild type normal-fed mice. However, the microglia of LC-fed GFAP-IL6 mice have significantly larger processes in length, which is observed in the peaks of the distributions of different morphological features. We call this “X at Y_max_”. Two-way ANOVA test.

**Figure 11:**
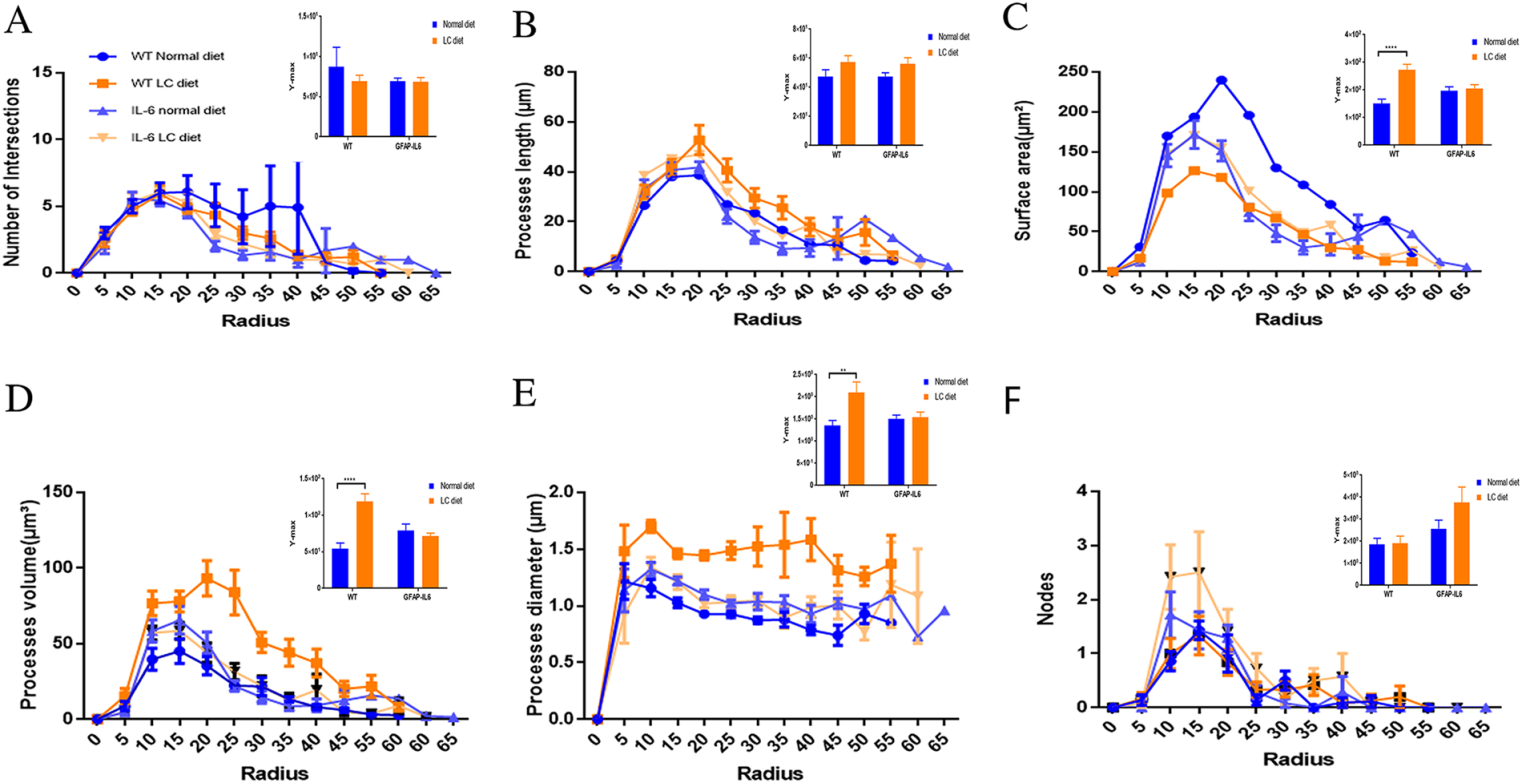
The effect of LC on microglial cells in Sholl analysis in the cerebellum region. (**A**–**F**) There was not any phenotype difference between wild type and GFAP-IL6 mice on a regular diet. However, we found significant differences between the wild type normal and LC-fed mice in terms of the microglial cells having a significantly larger surface area, process volume, and process diameter, which was observed in the peaks of the distributions of different morphological features. We call this “X at Y_max_”. Two-way ANOVA test.

In order to further the morphological characteristics of microglial cells from the hippocampus and the cerebellum, a quantitative analysis termed as a Sholl analysis was applied which revealed some further variation in morphology between wild type and GFAP-IL6 normal-fed and LC-fed mice. In the hippocampus, there was no significant difference between wild type and GFAP-IL6 normal-fed mice observed in any parameter. The microglia of wild type LC-fed mice has a significantly larger surface area (*p* < 0.0001), process volume (*p* < 0.0001), and process diameter (*p* < 0.0018) compared to that of wild type normal-fed mice. However, the microglia of LC-fed GFAP-IL6 mice have significantly larger processes in their length (*p* < 0.018) compared to GFAP-IL6 normal-fed mice **(**Fig. 10A–F). In the cerebellum, there was not any genotype difference between wild type and GFAP-IL6 normal-fed mice. In wild type fed mice, the microglial cells have a significantly larger surface area (*p* < 0.0001), process volume (*p* < 0.0001), and process diameter (*p* < 0.0037) than that of wild type normal-fed mice (Fig. 11A–F).

**Figure 12:**
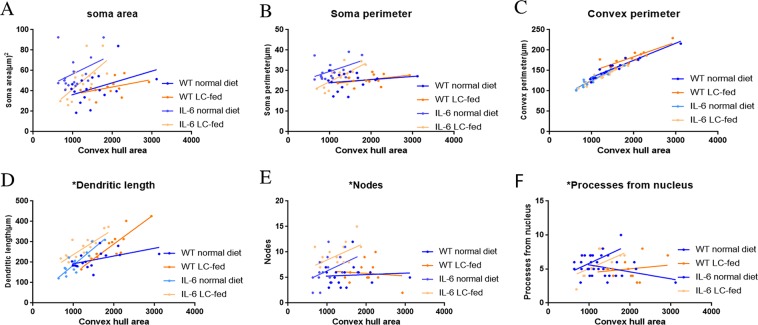
Bivariate correlation analysis of overall microglial cell size with each morphological characteristic in the hippocampus showing significant correlation in LC-fed GFAP-IL6 mice. (**A**–**F**) Different morphological parameters in each cohort have been plotted against the convex hull area, showing significant correlation with the soma area, soma perimeter, number of nodes and primary dendrites in LC-fed GFAP-IL6 mice and a convex perimeter and increase in dendritic length of each cohort with the increasing size of the entire cell.

**Figure 13:**
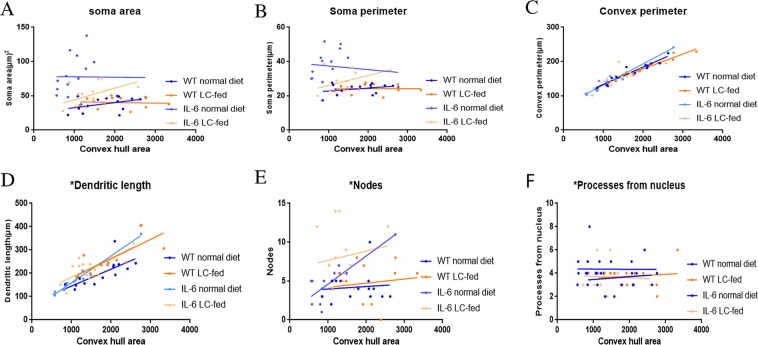
Bivariate correlation analysis of overall microglial cell size with each morphological characteristic in cerebellum. (**A**–**F**) Different morphological parameters in each cohort have been plotted against the convex hull area which shows significant correlations in all cohorts in the convex perimeter and dendritic length with overall cell size. However, the numbers of nodes in GFAP-IL6 non-fed cohorts increased with the overall increase in microglial cells.

In the hippocampus, a correlation analysis of overall microglial cell size with each morphological characteristic revealed a significant correlation with the soma area, soma perimeter, number of nodes and primary dendrites in LC-fed GFAP-IL6 mice, which clearly revealed that the microglial cells of the GFAP-IL6 LC-fed mice potentially increased the overall soma size and number of dendrites. In addition, the convex perimeter and dendritic length of each cohort increased with the increasing size of the entire cell. However, the rest of the parameters in the other cohorts remained consistent **(**Fig. 12A–F, Table 3**)**. In the cerebellum, significant correlations were observed in all cohorts in convex perimeter and dendritic length with overall cell size, showing that the convex perimeter and the length of dendrites increased with the overall increase in microglial cells. Moreover, the numbers of nodes in GFAP-IL6 non-fed cohorts were increased with the overall increase in microglial cells. The other parameters remained consistent across the cohort, regardless of the cell size **(**Fig. 13A–F, Table 4).

**Table 3:** Bivariate correlation of morphological characteristics with overall microglial cell size in the hippocampus in all the cohorts.

	Soma area		Soma perimeter		Convex perimeter		Dendritic length		Nodes		Primary dendrites	
	R^2^	Correlation	R^2^	Correlation	R^2^	Correlation	R^2^	Correlation	R^2^	Correlation	R^2^	Correlation
WT NF	0.17	0.10	0.04	0.44	0.91	**** < 0.0001	0.26	*0.04	0.005	0.786	0.21	0.06
WT LC	0.17	0.17	0.16	0.18	0.70	***0.0006	0.77	***0.0001	0.010	0.749	0.02	0.59
GFAP-IL6 NF	0.13	0.15	0.22	0.06	0.90	**** < 0.0001	0.80	**** < 0.0001	0.161	0.122	0.23	0.05
IGFAP-IL6 LC	0.40	**0.008	0.51	**0.002	0.92	**** < 0.0001	0.58	***0.0005	0.247	*0.05	0.28	*0.03

**Table 4:** Bivariate correlation of morphological characteristics with overall microglial cell size in the cerebellum.

Cohorts	Diet	Soma area(µm)^2^ (Mean ± SEM)	Soma perimeter(µm) (Mean ± SEM)	Convex 2D (area) (µm)^2^(Mean ± SEM)	Convex perimeter(µm) Mean ± SEM)	Total length(µm) (Mean ± SEM)	Number of nodes (Mean ± SEM)	Number of dendrites (Mean ± SEM)
WT	Normal food	15.88 ± 1.46	16.52 ± 0.79	885.3 ± 76.14	119.3 ± 5.82	163.2 ± 10.07	4 ± 0.45	4.81 ± 0.29
WT	LC	21.22 ± 4.13	18.46 ± 1.35	1005 ± 79.18	129.7 ± 5.25	167.9 ± 8.34	4 ± 0.40	4.87 ± 0.23
GFAP-IL6	Normal food	14.68 ± 2.13	15.78 ± 1.14	1418 ± 128.3	147.9 ± 6.01	275 ± 17.8	7.62 ± 0.77	6.06 ± 0.51
GFAP-IL6	LC	16.66 ± 1.99	16.66 ± 1.07	968.3 ± 74.04	125.8 ± 4.24	175.2 ± 14.54	5.43 ± 0.56	4.06 ± 0.23

### Effect of LC curcumin on astrocytes morphology in the hippocampus

**Figure 14:**
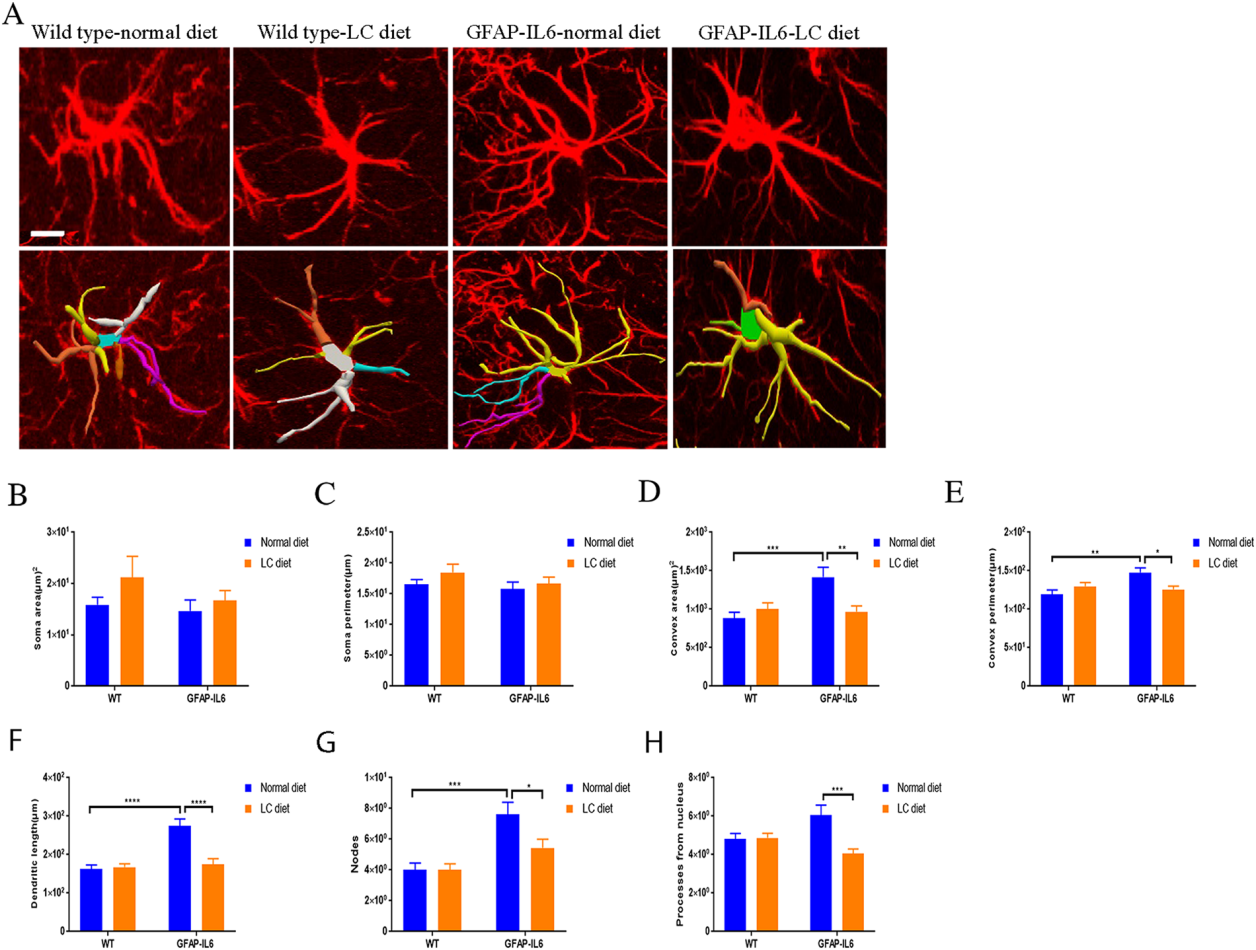
Effect of LC on astrocyte morphology in the hippocampus showing significant decrease in overall astrocytes size in LC-fed GFAP-IL6 mice. (**A**) 3D reconstruction of astrocytes in wild type and GFAP-IL6 in both showing that the LC diet treated and non-treated mice significantly decreased the dendritic length, number of processes, convex area, convex perimeter, and number of nodes Scale bar 10 µm. (**B**–**H**) Graphs showing the morphological changes in Iba-1^+^ microglia. Two-way ANOVA, Tukey’s post-test, mean ± SEM).

In inflammation, astrocytes are activated like microglial cells and undergo cellular hypertrophy which leads to the alteration of their size and shape. We, therefore, characterized the morphology of astrocytes in the hippocampus of wild type normal-diet, wild type LC-diet, GFAP-IL6 normal-diet, and GFAP-IL6 LC-diet mice **(**Fig. 14). In both wild type normal-diet and wild type LC-diet, we observed almost similar astrocytes and there were no significant differences. However, the astrocytes in the GFAP-IL6 normal-diet mice had a significantly larger dendritic length (275 ± 17.8 µm), number of processes (6.06 ± 0.51), convex area (1418 ± 128.3 µm^2^), convex perimeter (147.9 ± 6.01 µm) and number of nodes (7.62 ± 0.77) compared to the wild types. The LC diet significantly decreased the dendritic length (175.2 ± 14.54 µm) (*p* < 0.0001), number of processes (4.06 ± 0.23) (*p* < 0.0006), convex area (968.3 ± 74.04 µm^2^) (*p* < 0.0056), convex perimeter (125.8 ± 4.24 µm) (p < 0.026), and number of nodes (5.43 ± 0.56) (*p* < 0.041) **(**Table 5**)**.

**Table 5:** Morphological analysis of GFAP^+^ astrocytes in the hippocampus.

	Soma area		Soma perimeter		Convex perimeter		Dendritic length		Nodes		Primary dendrites	
	R^2^	Correlation	R^2^	Correlation	R^2^	Correlation	R^2^	Correlation	R^2^	Correlation	R^2^	Correlation
WT NF	0.15	0.16	0.09	0.28	0.91	**** < 0.0001	0.53	**0.0028	0.008	0.7567	0.012	0.6987
WT LC	0.006	0.80	0.08	0.99	0.94	**** < 0.0001	0.54	**0.0059	0.01	0.6701	0.013	0.7196
GFAP-IL6 NF	0.0001	0.96	0.01	0.65	0.94	**** < 0.0001	0.96	**** < 0.0001	0.68	***0.0003	0.014	0.9854
GFAP-IL6 LC	0.204	0.12	0.19	0.13	0.62	**0.0014	0.49	**0.0075	0.02	0.6396	0.002	0.8734

**Figure 15:**
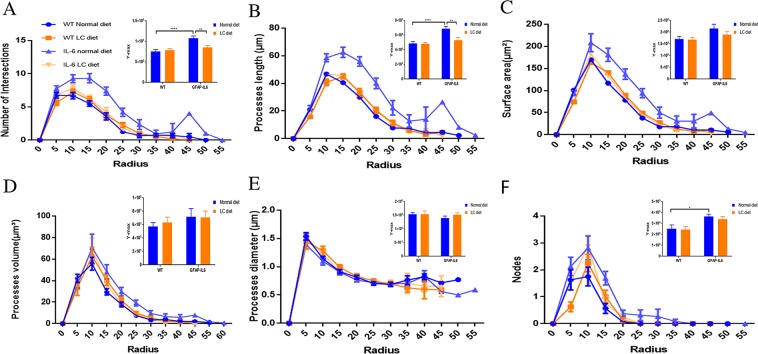
LC increased the ramification of astrocytes in the hippocampus region. (**A**–**F**) A Sholl analysis confirmed a significant decrease in a number of intersections and processes length in LC-fed GFAP-IL6 mice compared to normal food-fed GFAP-IL6. No significant changes occurred in wild type fed mice. LC significantly affects the inflamed astrocytes, which is observed in the peaks of the distributions of different morphological features being closer to the center compared to normal food-fed GFAP-IL6 mice. We call this “X at Y_max_”. Two-way ANOVA test.

Furthermore, we quantified the astroglial cells by Sholl analyses. In the same manner, this analysis did not reveal any significant changes between wild type normal-fed and LC-fed mice. We found that the LC significantly reduced the number of intersections and the length of processes compared to the normal-diet fed GFAP-IL6 mice **(**Fig. 15A–H).

**Figure 16:**
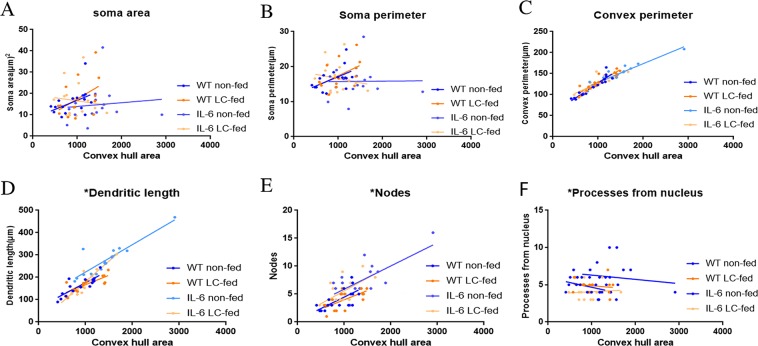
Bivariate correlation analysis of overall astrocyte size with each morphological characteristic in hippocampus. (**A**–**F**) Different morphological parameters in each cohort have been plotted against the convex hull area which revealed that the convex perimeter, dendritic length and the number of nodes increased significantly with the overall increase in the entire cell structure in LC-fed animals.

We performed the bivariate correlation of morphological characteristics with overall astroglial cell size to investigate the impact of the size of astroglial cells on the structural parameters. We observed some positive correlation of the astroglial cell size with some of the morphological parameters. The data revealed that the convex perimeter, dendritic length and the number of nodes increased significantly with the overall increase in the entire cell structure **(**Fig. 16A–H, Table 6).

**Table 6:** Bivariate correlation of morphological characteristics with overall astroglial cell size in the hippocampus.

	Soma area		Soma perimeter		Convex perimeter		Dendritic length		Nodes		Primary dendrites	
	R^2^	Correlation	R^2^	Correlation	R^2^	Correlation	R^2^	Correlation	R^2^	Correlation	R^2^	Correlation
WT non-fed	0.19	0.08	0.25	*0.04	0.86	**** < 0.0001	0.70	**** < 0.0001	0.47	**** < 0.0001	0.11	0.20
WT LC-fed	0.21	0.08	0.25	0.05	0.84	**** < 0.0001	0.63	****0.0002	0.41	***0.0002	0.02	0.55
IL-6 non-fed	0.01	0.69	0.0001	0.96	0.89	**** < 0.0001	0.78	**** < 0.0001	0.46	**** < 0.0001	0.02	0.59
IL-6 LC-fed	0.01	0.68	0.02	0.54	0.69	**** < 0.0001	0.87	**** < 0.0001	0.36	**** < 0.0001	0.008	0.73

### LC feeding improved the levels of synaptophysin in the GFAP-IL6 mice

In order to investigate whether LC fed wild type and GFAP-IL6 mice showed any changes in synaptic markers expression compared to the normal diet-fed wild type and GFAP-IL6 mice at 8 months of age, the presynaptic marker synaptophysin and the postsynaptic marker PSD95 were quantified in cerebellum using Western blots.

**Figure 17:**
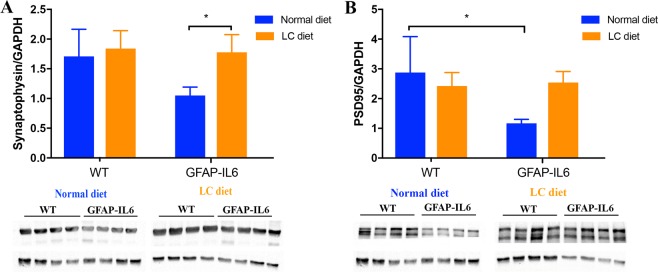
LC feeding in the GFAP-IL6 mice restored the levels of presynaptic protein, synaptophysin to that of the wild type animals. Pre- and postsynaptic protein levels measured by Western blots in the wild type and GFAP-IL6 normal and LC-fed cohorts revealed that LC was able to restore the levels of synaptophysin to those of the wild type (*p < 0.05).

For synaptophysin, a significant difference was found between the groups [F (3, 12) = 5.268, *p* = 0.001]. We found that synaptophysin levels of LC-fed GFAP-IL6 mice (1.78 ± 0.15) was significantly higher than the normal diet fed GFAP-IL6 mice (1.05 ± 0.07) (p = 0.03), restored to the level of the wild type mice. The levels of synaptophysin in LC-fed wild type mice (1.84 ± 0.15) were slightly higher compared to the normal diet fed wild type (1.71 ± 0.23) **(**Fig. 17A).

For PSD95, a significant difference was found between the groups [F (3, 12) = 4.854, *p* = 0.02]. A 59% of loss of PSD95 was observed in the normal diet-fed GFAP-IL6 mice (1.17 ± 0.07) compared to the normal diet-fed wild type (2.87 ± 0.61). These levels were found to increase in the LC-fed GFAP-IL6 mice (2.54 ± 0.19), but no significance was observed between the groups **(**Fig. 17B).

### LC improved motor endurance loss measured by the Rotarod test in the GFAP-IL6 male mice

In order to investigate whether LC is able to ameliorate the motor function loss of the GFAP-IL6 mice we measured their performance on the Rotarod, which measures motor balance and muscle strength.

**Figure 18:**
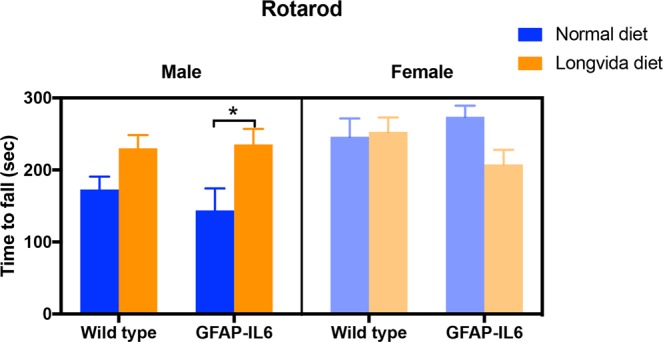
Rotarod performance is positively affected by the LC diet in the GFAP-IL6 male animals. Diet had a significant impact on the male animals when we measured their rotarod performance [F(1,32) = 10.7, *p* = 0.03], multiple comparisons showing a significant difference in the GFAP-IL6 normal versus LC diet groups (*p < 0.05).

We examined the results by separating males and females and using 3 way ANOVAs at first. We found a significant main effect of gender [F(1,111) = 7.296, *p* = 0.008], so we kept our analysis gender specific. For example, female GFAP-Il6 mice on normal diet were able to stay on the Rotarod for an average of 73 seconds (42%) longer than their male counterparts (Fig. 18).

LC supplementation in the male GFAP-IL6 mice improved their performance on the Rotarod. Multiple comparisons showing a significant difference in the GFAP-IL6 normal diet versus the GFAP-IL6 LC diet groups, with LC fed male GFAP-Il6 mice being able to stay an average of 92 seconds (63%) longer on the Rotarod than their non-supplented counterparts (*p* < 0.05) [F(1, 32) = 10.7, *p* = 0.03] (Fig. 18**)**. We found no significant differences between the female control or LC fed GFAP-IL6 mice.

## Discussion

While it has been suggested that cytokine-suppressive anti-inflammatory drugs (CSAIDs) such as curcumin are able to downregulate chronic glial activation and protect against inflammation-induced neuronal damage in AD^31^, there have been relatively few investigations on how CSAIDs could potentially reduce chronic neuroinflammatory processes in preclinical settings. Identification of the effects of CSAIDs on chronic neuroinflammation will facilitate the development of further anti-inflammatory medication to be potentially used in neurodegenerative diseases.

Despite its various pharmacological and therapeutic activities, the instability of curcumin at physiological pH and its susceptibility to intestinal and hepatic metabolism have limited its therapeutic use. Although curcumin is well absorbed in glucuronidated and sulfated forms by the intestine and liver, which increases hydrophobicity, it therefore limits blood-brain barrier permeability and lowers bioavailability to the brain^32,33^. However, free curcumin is membrane permeable because it is lipophilic and fulfills the Rule of 5 characteristics of blood-brain barrier permeable molecules. To decrease the glucuronidation of curcumin, the use of piperine has been recommended^34^. To increase its stability and subsequent bioavailability, a number of different encapsulation approaches have been employed including the use of liposomal curcumin, including Longvida (LC) curcumin.

LC is a solid lipid curcumin particle form of dietary curcumin with high bioavailability and absorption, and in this study it has been used in the GFAP-IL6 mouse model, to investigate its therapeutic effects in chronic neuroinflammation. In the GFAP-IL6 mouse model, the murine IL-6 gene is expressed by astroglia under the transcriptional control of the murine glial fibrillary acidic protein (GFAP) promoter, resulting in cytokine-induced low grade chronic glial activation, astrogliosis, and microgliosis^22^. Using this model, we have assessed the anti-inflammatory effect of LC after 4 months of feeding and compared the results with normal diet-fed animals, using both wild type and GFAP-IL6 animals on both diets.

The present study concluded that the number of Iba-1^+^ microglia is significantly increased in both the hippocampus and the cerebellum in GFAP-IL6 normal-fed mice compared to the wild type normal-fed mice, which is in accordance with our previous report^25^. LC feeding significantly decreased the Iba-1^+^ microglia numbers by 25% in the hippocampus and by 48% in the cerebellum in the GFAP-IL6 animals. These reductions in the activated microglia numbers could indicate overall decreased levels of glial activation, meaning an improvement in the condition associated with the neuroinflammatory process^35^. In accordance with our results, a previous study conducted in a mouse model of AD has compared the effect of solid lipid curcumin particles (SLCP) and normal curcumin. They reported that both SLCP and curcumin significantly reduced the Iba-1^+^ microglia numbers compared to the control^36^.

In the present study, we have investigated some of the morphological changes between ramified and non-ramified microglia. When investigating the hippocampus, we found that the Iba-1^+^ microglial cells of GFAP-IL6 mice normal-fed had significantly larger soma areas, small convex areas, and convex perimeter than those of wild type. The LC diet had significantly increased the dendritic length and number of nodes than that of GFAP-IL6 normal-fed mice. However, in the cerebellum, Iba-1^+^ microglial cells of GFAP-IL6 normal-fed mice had a significantly large soma area, soma perimeter and a smaller convex area and convex perimeter than those of the wild type non-fed mice. The LC diet had significantly reduced the soma area, soma perimeter and high number of nodes of the Iba-1^+^ microglial cells of GFAP-IL6 LC-fed mice compared to that of GFAP-IL6 normal-fed mice. Comparing our results to other studies, we found that a previous study performed on healthy adult mice has reported that ramified cells have small somas, and long and thin processes, which are necessary for active surveillance of microglia microdomains^37^. However, when microglial cells are activated in response to the neuronal assault, injury or inflammation, they undergo morphological transformations, such as increased soma size and thickening of the processes^8^.

A recent study performed in mice has proved that solid lipid curcumin particles can reduce the aggregation and de-ramification of microglial cells in the hippocampus^36^, which is consistent with previous reports.

Our study has reported that LC has the potential to downregulate the TSPO^+^ microglia in chronic neuroinflammation. A recent study performed in the same mouse model found that the number of TSPO^+^ cells increased in GFAP-IL6 mice and it increased even more in Tenilsetam treated mice^30^. The present study has revealed that the TSPO^+^ microglia is significantly increased in both regions (hippocampus and cerebellum) of the brain in GFAP-IL6 normal-fed mice compared to the wild type normal-fed mice. After feeding the LC to the mice, the TSPO^+^ microglia numbers significantly reduced by 31% in the cerebellum and 24% in the hippocampus.

However, microglia were also shown to have neuroprotective properties, for instance in AD, and so reducing the numbers might worsen the disease^38^. Therefore, microglia numbers alone, without the rest of the pathological improvement, does not mean decreased chronic glial activation. Because of this, we also examined the motor functions of our cohorts and found that long-term LC supplementation improved the balance and motor coordination on the rotarod for the male GFAP-IL6 mice. Motor function impairment GFAP-IL6 mice in the rotarod test have been previously reported by our group^25^. IL-6 mediated motor function impairment has also been found in mouse models taking the rotarod test following experimental traumatic brain injury (TBI)^39^. Similar studies have provided evidence for IL-1 and TNF-α mediated motor function deficits in mice subjected to TBI^40^. Impaired rotarod performance has also been reported in mice with astrocyte-derived IL-3 expression in the brain^41^. Other studies have also found similar improvements in motor skills measured by the rotarod test after curcumin supplementation. For example, curcumin feeding of 50, 100 and 200 mg/kg per os for 3 weeks in a mouse model of Parkinson’s disease, has shown to ameliorate rotarod test deficits in a dose-dependent manner compared to non-treated counterparts^42^. Daily oral feeding of mice with the inherited peripheral neuropathy model with curcumin in 50 or 100 mg per kg bodyweight doses for 90 days has revealed improved rotarod performance compared to non-fed counterparts^43^. Single intrastriatal injection of a mouse model for brain oxidative damage with curcumin (10–100 µg) or Theracurmin (30–300 µg) revealed improved rotarod performance of mice by both forms of curcumin in a dose-dependent manner^44^. However, single oral feeding of mice for the same model with curcumin (30–300 mg/kg) or Theracurmin (0.3–3 g/kg) improved rotarod performance in only the Theracurmin group.

This study reports that LC decreases the GFAP^+^ astrocytes and ultimately chronic neuroinflammation. A study performed in a mouse model of AD counted the number of GFAP^+^ cells in different brain areas, specifically in the CA1, the CA3, the dentate gyrus, the subiculum, the entorhinal cortex, and the striatum. The authors observed inhibition of GFAP^+^ cell expression in the normal curcumin and SLCP-treated mice^36^. This is consistent with our findings which showed that, in the hippocampus, GFAP-IL6 mice had a significantly larger number of GFAP^+^ astrocytes compared to wild type mice and in the LC-fed GFAP-IL6 mice, a significant reduction in GFAP^+^ astrocytes were observed by 30% compared to the GFAP-IL6 normal food-fed mice. A recent study using curcumin formulation reported that curcumin treatment decreased astrocyte activation^36^. Herein, a morphological assessment of astrocytes in the hippocampus shows that GFAP-IL6 normal-diet mice have a significantly larger dendritic length, number of processes, convex area, convex perimeter and number of nodes compared to the wild types. The LC diet significantly decreases the dendritic length number of processes, convex area, convex perimeter and number of nodes.

It has been shown that an increase in activated microglia leads to synaptic damage^45,46^, further leading to dysfunctional synaptic transmission in neurodegenerative diseases, measured by a significant loss of the pre- and post-synaptic proteins in the inflammatory state. Our results support this finding by estimating the levels of synaptic proteins (synaptophysin, PSD95) in the cerebellum of wild type and GFAP-IL6 mice (both normal-fed and LC-fed). We found that after LC treatment, 42% of synaptophysin and 59% of PSD95 protein levels were restored in the cerebellum of GFAP-IL6 mice. In correspondence with our results, others have also found that using curcumin on the APPswe/PS1dE9 double transgenic mouse model restores the quantity and structure of the synapses measured by western blots for the PSD95 hippocampal CA1 area, with the curcumin treatment increasing the expression of this protein^47^.

In addition, we found that the female mice fed with LC had an increased bodyweight. This was probably due to the fact, that they had a baseline bodyweight somewhat higher than the other female cohort group, which was still within the normal weight range. We were satisfied with the weight gain of all the groups fed with LC, showing no signs of food or taste aversion.

With these reported results, our study has opened a window to further investigate this particular curcumin formulation and its mode of action on both microglia and astroglial activation. In the future, the effects of LC directly on cognitive decline and neuronal degeneration should be also investigated. The main potential limitation of this study is that it did not investigate the molecular mechanisms of the inhibition of glial cells, hence the signal transduction mechanism pathways in which curcumin could have carried out its effects on glial cells is still unknown.

In summary, this study investigated the impact of Longvida^®^ Optimised Curcumin formulation, against astro- and microglial cell activation and chronic neuroinflammation. We found that LC has the potential to ameliorate chronic microglia and astroglial activation by reducing increased glial cell numbers and restoring their morphology towards the resting/ramified state. This study has provided strong evidence that LC has the potential to reduce chronic activation of microglia and astrocytes in the brain, and might be beneficial for preventing neurodegenerative disorders

## Materials and Methods

### Animals

Wild type (C57BL/6) and GFAP-IL6 mice of mixed genders weighing 20–30 g were housed in the animal facility of the School of Medicine, Western Sydney University under a temperature-controlled environment, with a normal 12 h/12 h light/dark cycle at 23 °C, 60 ± 10% humidity, and provided with food and water *ad libitum*. The experimental procedures were approved by Western Sydney University Animal Care and Ethics Committee (approval ID: A11393) and carried out in accordance with the rules established by the National Health and Medical Research Council of Australia. Heterozygous GFAP-IL6 mice and their non-transgenic littermates (wild type C57BL/6) were used at the age of 2 months.

### Grouping of animals and feeding with LC food pellets

In this long-term feeding study, animals were randomly assigned to 4 groups: Wild type C57BL/6, GFAP-IL6 mice fed with control food pellets and GFAP-IL6, GFAP-IL6 mice fed with LC diet (Table 7). The Longvida curcumin (LC) food pellets were supplied by Verdure Sciences Inc.; Noblesville, IN. The pellets consisted of Longvida^®^ Solid Lipid Curcumin Particles (SLCP) with enhanced bioavailability. At the age of 2 months, the mice were put on feeding at one defined dose for the duration of 6 months. Animals were monitored, with their body weight and the amount of food consumed recorded. At the age of 8 months, the behavioral test was performed followed by perfusion for histology.

**Table 7:** Summay of the cohorts, type of diet and number of mice used.

Cohorts	Type of food	Number of mice used		
		Iba-1	GFAP	TSPO
WT	Normal food	5	6	3
GFAP-IL6	Normal food	7	6	6
WT	LC (500 ppm)	7	6	3
GFAP-IL6	LC (500 ppm)	7	6	6

### Evaluating body weight and motor function

Before the evaluation of motor function, we monitored the weight of the animals during the feeding and after the completion of the feeding protocol, respectively. To assess the coordination and balance in rodents, we used an accelerating, rotating 9 cm diameter rod apparatus (Ugo Basile, Biological Research Apparatus, Varese, Italy), and used “time to fall” as the readout. Each mouse was placed on the apparatus at a steady speed of 5 rpm. The speed was gradually increased from 5 rpm to 40 rpm over a period of 300 seconds, and the latency until the mouse fell off the rod was measured (with a maximum cut-off time of 300 secs). Each mouse was subjected to three trials a day with a 45-min inter-trial interval. Data presented were averaged of the best two scores across the three trials.

### Histology and tissue sample preparation

For histological analysis, the tissue samples were prepared from all experimental cohorts. Mice were anaesthetized with Pentobarbitone (30–50 mg/kg IP (20–40 min. of anesthesia) and transcardially perfused with 30 ml of 0.9% normal saline using a peristaltic pump, followed by 60 ml of 4% cold paraformaldehyde (Merck) (in 0.1 M phosphate buffer). Brains were harvested and post-fixed in 4% paraformaldehyde for 24 hrs at 4 °C, and then transferred to 30% sucrose (in 0.1 M PB solution) for cryoprotection. After the brains sank to the bottom of the container, they were embedded and frozen with 6% gelatin. Fifty micrometer thick coronal sections were cut in six series using a Leica CM 1950 cryostat.

### Immunohistochemistry

For bright field microscopy, immunohistochemistry assays were performed on every 6th section of the brains to identify microglial activation using microglia markers (Iba-1 and TSPO). All washing and incubation procedures were performed using 0.1 M PBS unless stated otherwise. The sections were washed 3 times and treated with 1% H_2_O_2_ before being incubated for 2 hrs in the blocking solution (2% goat serum) to block non-specific antigen binding sites. Then they were incubated in the primary rabbit-anti-Iba1 antibody (1:500, Wako, # 019-19741) and rabbit-anti-TSPO (1:500, Merck, #ABC139) solutions for two days at 4 °C and subsequently in the secondary antibody (1:200, biotinylated goat anti-rabbit IgG; Life Technologies, #656140) for 2 hrs. The sections were washed 3 times and ABC solution (1:250, Vector Laboratories, # PK-6100) was applied for 2 hrs. Sections were then incubated in the developer solution containing 0.4 mg/ml DAB and 0.0006% hydrogen peroxide until an optimal color developed. In the end, the sections were washed, mounted, dehydrated, coverslipped and images were captured with a Zeiss microscope (MBF Bioscience).

### Fluorescence microscopy

Immunofluorescent staining against GFAP was performed on every 6th section from the brains to identify astroglial activation using GFAP as an astroglial marker. The sections were washed with 0.1 M PBS in order to remove the gelatine and incubated in the primary rabbit-anti-GFAP antibody (1:500, Dako, #20023331) solution for one day at 4 °C followed by a fluorescence secondary antibody (1:200, goat anti-rabbit IgG, Alexa 488; Thermo Fisher Scientific, # 1853312) for 2 hrs. The sections were washed, mounted and coverslipped with a fluorescent mounting medium (Vector Laboratories, #H1400).

### Stereological counting

The estimated number of glial cells in both the cerebellum and the hippocampus was counted on Iba1, TSPO and GFAP stained sections using the Zeiss AxioImager M2 microscope equipped with MBF Biosciences StereoInvestigator^30^. The contour of the cerebellum and the hippocampus was first drawn under the 2.5× objective. The size of the counting frame was 100 × 100 μm for the wild type in both the hippocampus and the cerebellum, 60 × 60 μm for GFAP-IL6 non-fed mice as well as for LC mice. However, it was 80 × 80 μm in the case of TSPO counting. The counting grid was 1000 × 1000 μm for the cerebellum and 800 × 800 μm for the hippocampus for the entire cohorts. The guard zone was 1 μm at the top and the bottom of the sections. Microglia and astrocytes were plotted on the screen using a marker as the focus moved from the top to the bottom of the sections using a 63x oil objective. This led to the Gunderson coefficient error of less than 0.1 in all cases (m = 1).

### Three-dimensional reconstruction of astrocytes

Samples were prepared as described above and the images were taken using a confocal ZEISS laser scanning microscope (LSM-5) with an argon laser and processed using the Zen 2009 software package. Z-stacks were captured using a 20 × objective, NA1.0 for reflective imaging, at a step size of 0.1um (unless specified otherwise). Reflective imaging was achieved using the 488 nm wavelength. For the three-dimensional reconstruction of astrocytes, Neurolucida 360 (MBF Bioscience) software was used. To better identify the objects and provide greater accuracy, all the images were taken using a 20× high power objective with the laser scanning confocal microscope. A total of 16–20 cells in the hippocampus of the brain were traced in each experimental cohort and fully or partially reconstructed (only soma). After importing Z-stacks to Neurolucida 360 software, astrocytes were manually reconstructed along required planes, getting a 3D image of each cell.

### Analysis of reconstructed cells

Morphometric data of each astrocyte were extracted by the software and thus, each reconstructed cell was subject to multiple parameters. The soma area, soma perimeter, convex 2D area, convex perimeter, total length of all processes, total volume of all processes, total density and number of nodes (branch points) and dendrites of all the processes were measured. In order to determine changes in the size of the cells in relation to distance from the cell soma, a Sholl analysis was performed for each astroglial cell. Z-stack images of live astrocytes were condensed into a maximum intensity and the Sholl analysis was applied in Neurolucida 360 software. Concentric circles (radii) originating from the soma were spaced 5 μm apart in this case. This analysis determined the number of intersections, process length (µm), the surface area of the cells (µm^2^), process volume (µm^3^), process diameter (µm) and the number of nodes of the cells for each radius.

### Western blot

Western blot experiments were performed as described elsewhere^25^. In short, tissue samples from the cerebellum of mice brain were homogenized (10% w/v) in an ice-cold lysis buffer [100 mM Tris (pH 7.4), 150 mM NaCl, 1 mM EDTA, 1 mM EGTA, 1% Triton-x 100 (v/v) and 0.5% sodium deoxycholate] with freshly added protease (Thermo Fisher, Cat No: A32963) and phosphatase inhibitors (Sodium orthovanadate, sodium fluoride, staurosporine), using a motor-driven homogenizer (Thomas Scientific) on ice. The lysate was centrifuged at 14,000 rpm for 20 min at 4 °C and the total protein estimation of the supernatant was tested using the Bradford reagent (Bio-Rad) at 595 nm (UV-absorption). After protein quantification, tissue extracts (25 μg) were boiled in a Laemmli sample buffer at 95 °C for 10 mins, followed by 10% SDS-PAGE electrophoresis at 90 V for 1.5 hours, electro-transferred to nitrocellulose membranes for 2 hours at 100 V. Non-specific protein binding was blocked with 5% milk in 0.1% Tween TBS. Membranes were incubated overnight at 4 °C with a mouse monoclonal antibody to PSD95 (0.5 µg/mL, # MABN68, Merck) or a mouse polyclonal antibody to Synaptophysin (1:500, # ab8049, Abcam) in 0.1% Tween TBS. Membranes were then washed and incubated for 1 hour with an HRP-conjugated secondary antibody (1:10,000) in 0.1% Tween TBS. Membranes were then washed and immunoreactivity was visualized with a chemiluminescence detection method (ECL, Merck Millipore) using Imagelab software (Biorad). Optical densitometry was performed on each targeted band (Synaptophysin: 38 kDa; PSD-95: 95 kDa) using the computerized image analysis software ImageJ (NIH).

### Statistical analysis

For all our behavioral analysis, at first a three-way ANOVA was used with the variable factors “age,” “genotype,” and “gender.” We did not detect any sex-effects (all *p* > 0.05) and therefore combined male and female data sets for further analysis using two-way ANOVA for “genotype” and “age” with Bonferroni correction for multiple comparisons. In line with Rothman^48^ and Perneger^49^, the data were not adjusted for multiple comparisons and were interpreted as such in the discussion. In the case of significant interactions between “genotype” and “age”, we split data by the corresponding factor. Histological quantification analysis results were analyzed by using two-way ANOVA with Bonferroni correction for multiple comparisons. Differences were regarded as statistically significant when *p* < 0.05. [(DFn, DFD) = F, *p]* indicating the value of the F-test, the degrees of freedom of the numerator and denominator and the significance, respectively, and were used to determine whether the factors significantly affected the result. A three-way ANOVA analysis was performed using SPSS 22.0 for Windows and a two-way ANOVA with multiple comparisons were performed with a Prism GraphPad (version 7). Statistical outliers were defined where the values were smaller or bigger than ±2.5 times of standard deviations from the overall mean. Western blot results were analyzed using one-way ANOVA. Results are presented as mean ± SEM.

## Supplementary information

Supplementary figure 1.

## Data Availability

All data and materials described in the manuscript, including all relevant raw data, will be freely available to any scientists upon request from the corresponding author. Minimal (avaraged from raw data) data sets are included within the manuscript.
