# Supplementary material for: Effects of a solid lipid curcumin particle formulation on chronic activation of microglia and astroglia in the GFAP-IL6 mouse model
Source: Sci Rep. 2020 Feb 11;10:2365. doi: 10.1038/s41598-020-58838-2 (PMC7012877; doi:10.1038/s41598-020-58838-2)
Supplement: Supplementary file 1 — Supplementary figure 1. [file 41598_2020_58838_MOESM1_ESM.pdf]

Effects of a solid lipid curcumin particle formulation on chronic activation of microglia and astroglia in the GFAP-IL6 mouse model

Faheem Ullah, Rustam Asgarov, Madhuri Venigalla, Huazheng Liang, Garry Niedermayer, Gerald Münch, Erika Gyengesi

Supplementary figure 1. Whole gel images of both PSD95 and Synaptophysin

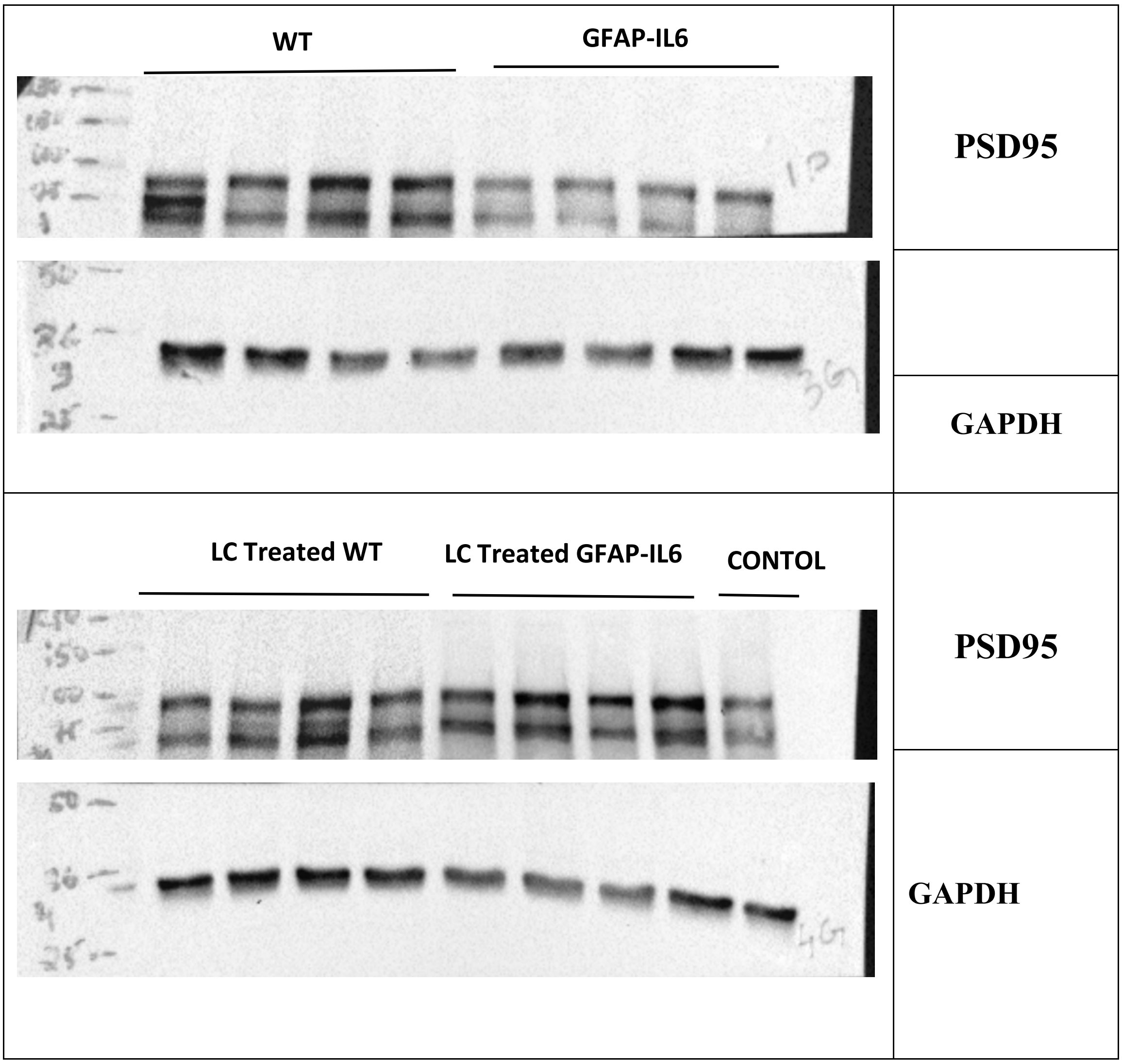

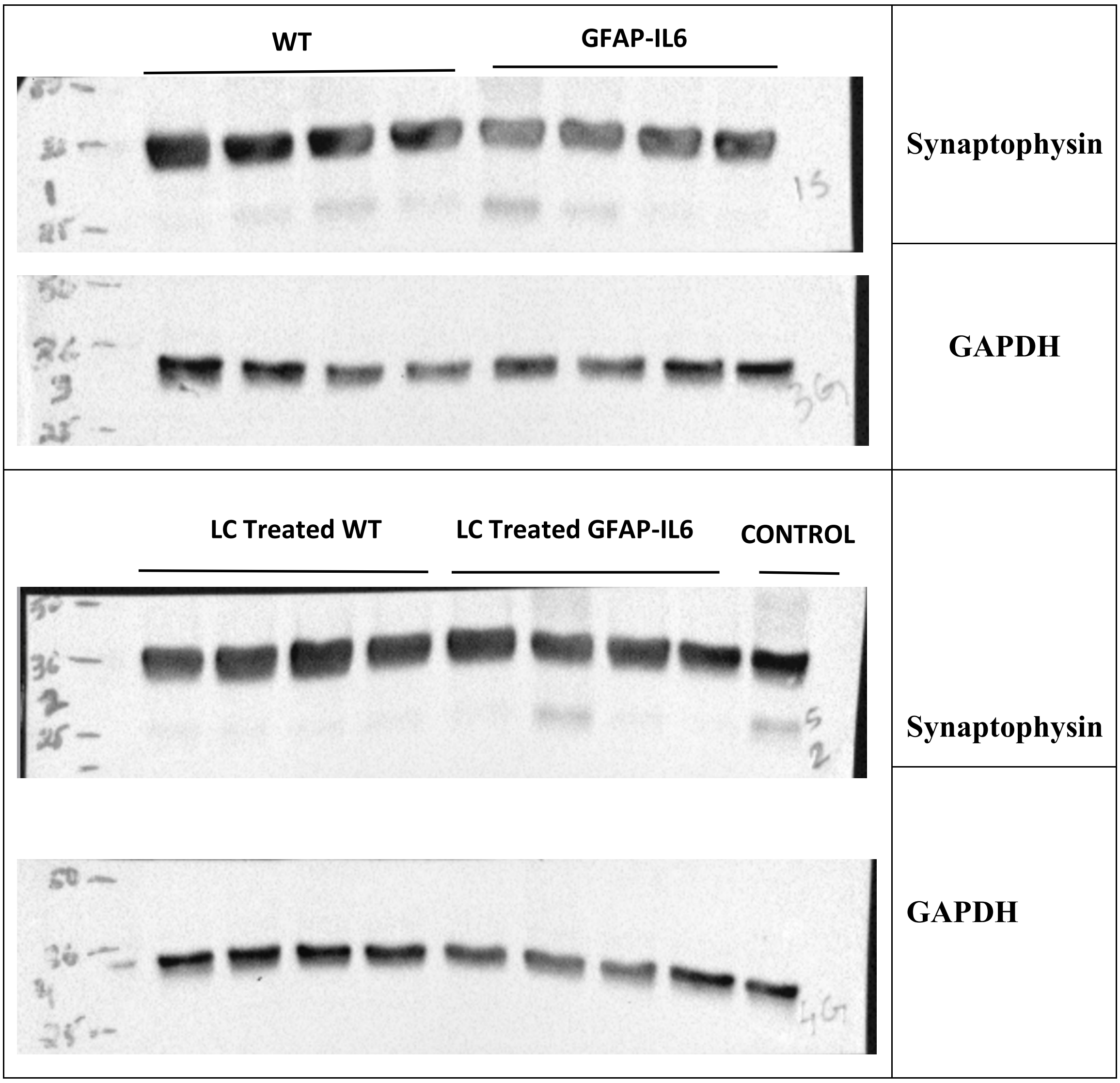
WT
GFAP-IL6
Synaptophysin
GAPDH
LC Treated WT
LC Treated GFAP-IL6
CONTROL
Synaptophysin
GAPDH
